# Increased spatial and temporal autocorrelation of temperature under climate change

**DOI:** 10.1038/s41598-018-33217-0

**Published:** 2018-10-04

**Authors:** Grace J. Di Cecco, Tarik C. Gouhier

**Affiliations:** 1 Northeastern University, Department of Biology, 360 Huntington Ave., Boston, MA 02115 USA; 2 Northeastern University Marine Science Center, 430 Nahant Rd., Nahant, MA 01908 USA

## Abstract

Understanding spatiotemporal variation in environmental conditions is important to determine how climate change will impact ecological communities. The spatial and temporal autocorrelation of temperature can have strong impacts on community structure and persistence by increasing the duration and the magnitude of unfavorable conditions in sink populations and disrupting spatial rescue effects by synchronizing spatially segregated populations. Although increases in spatial and temporal autocorrelation of temperature have been documented in historical data, little is known about how climate change will impact these trends. We examined daily air temperature data from 21 General Circulation Models under the business-as-usual carbon emission scenario to quantify patterns of spatial and temporal autocorrelation between 1871 and 2099. Although both spatial and temporal autocorrelation increased over time, there was significant regional variation in the temporal autocorrelation trends. Additionally, we found a consistent breakpoint in the relationship between spatial autocorrelation and time around the year 2030, indicating an acceleration in the rate of increase of the spatial autocorrelation over the second half of the 21^st^ century. Overall, our results suggest that ecological populations might experience elevated extinction risk under climate change because increased spatial and temporal autocorrelation of temperature is expected to erode both spatial and temporal refugia.

## Introduction

Although classical studies have focused almost exclusively on determining the ecological impacts of shifting average environmental conditions, there is growing recognition that statistical properties beyond the mean can play an equally important role in shaping the structure and functioning of ecosystems under climate change^1–3^. For instance, high variance in an environmental variable can destabilize populations by inducing greater fluctuations in their abundance over time and thus increase the risk of stochastic extinction^1,2,4^. Even when environmental variance is too weak to promote extinction risk, it can have a more insidious effect on ecological systems due to nonlinear averaging. Specifically, if a population responds nonlinearly to environmental conditions, then changes in the temporal or spatial properties of the environment (e.g., temporal or spatial variance) can alter the mean ecological response even if the mean environmental conditions remain constant, and the strength of this effect will depend on the degree of nonlinearity in the functional response of the population^1,5,6^. This implies that the variance of an environmental variable has consequences for ecological systems that cannot be inferred from their average values alone^1^. Indeed, studies have shown that variation in environmental variables impacts natural populations and consideration of variance can be required for successful model predictions^5^. Hence, in order to determine how climate change will impact ecological systems, it is at least as important to study variation in temperature and other critical environmental variables as it is to study their mean.

In addition to variance, it is also crucial to consider the temporal structure or autocorrelation of environmental variables such as temperature, as the variation in environmental variables is predicted to change over time^6^. Studies of historical temperatures have shown an increase in their temporal autocorrelation from 1961 to 1990^2,3^, and these trends are expected to increase under climate change^5,7^. Quantifying such changes in the temporal autocorrelation of environmental variables is critical in order to accurately predict the dynamics and persistence of ecological systems under climate change. Indeed, increased temporal autocorrelation in climate variables has been linked to lower persistence of model and natural populations^3,8^. In general, theory predicts that a reddening of environmental time series (an increase in its temporal autocorrelation) can extend the duration of poor conditions and thus promote extinction risk by eroding temporal refugia or rescue effects^9,10^.

The combination of increased temporal variance and autocorrelation in the environment could have even more dire consequences for ecological systems. For example, Dillon *et al*. showed that adding power to lower frequencies of temperature time series increased their temporal variance and autocorrelation, and thus the overall incidence and persistence of long-term heat waves and cold snaps^2^. Such increased intensity (temporal variance) and duration (temporal autocorrelation) of harsh conditions can promote extinction risk. Hence, the destabilizing effect of increased temporal environmental variability can be amplified by an increase in its autocorrelation. Overall, this suggests that increased temporal variance and autocorrelation of environmental conditions may interact synergistically to destabilize ecological systems.

The link between spatial autocorrelation of the environment and population dynamics has also been well established, with theoretical and empirical studies showing that spatiotemporally correlated or synchronized fluctuations in environmental conditions can give rise to synchronized fluctuations in ecological populations (i.e., the Moran effect^11,12^). For instance, increased spatial synchrony in air temperature due to changes in basin-scale climate forcing (North Atlantic Oscillation) gave rise to increased synchrony in Greenland animal populations^1,3,6,13,14^. In general, increased spatial autocorrelation in the environment is expected to promote the scale and the magnitude of synchronized fluctuations in populations and thus reduce the spatial heterogeneity of ecological systems. In doing so, increased spatial environmental autocorrelation can thus promote extinction risk at local and regional scales by eliminating the potential for spatial rescue effects between interconnected but increasingly synchronized populations^15–18^.

Although the negative effects of spatial and temporal autocorrelation have been established independently in model and natural systems, little is known about how these two phenomena will interact under climate change. Overall, theory suggests that increased spatial and temporal autocorrelation of environmental factors like temperature are expected to interact synergistically to destabilize ecological communities by disrupting both temporal and spatial rescue effects^4^. To determine the potential for such synergistic interactions, we characterized spatial and temporal autocorrelation in temperature from climate model projections. This was accomplished by analyzing temperature data from 1871 to 2099 using spectral analysis to measure temporal autocorrelation by calculating the spectral exponent and estimating spatial autocorrelation by determining the lag distance at which temperature becomes spatially uncorrelated (i.e., the spatial range).

We found that temporal and spatial autocorrelation increased over time in individual climate models and the multimodel mean. However, there was considerable regional variation in temporal autocorrelation, with western North America, central South America, northwestern Eurasia and North Africa seeing significant increases in temporal autocorrelation, but West Africa and the Southern Ocean seeing significant decreases in temporal autocorrelation. Piecewise regression analyses showed consistent breakpoints in spatial trends in autocorrelation around the year 2030 globally, and spatial trends in autocorrelation were statistically significant after the breakpoint across time windows of different sizes. Our results suggest that climate change is likely to increase the spatial and temporal homogeneity of temperature, which may negatively impact connectivity between source and sink populations and thus reduce the persistence of ecological communities.

## Results

### Global trends in temporal autocorrelation

**Figure 1:**
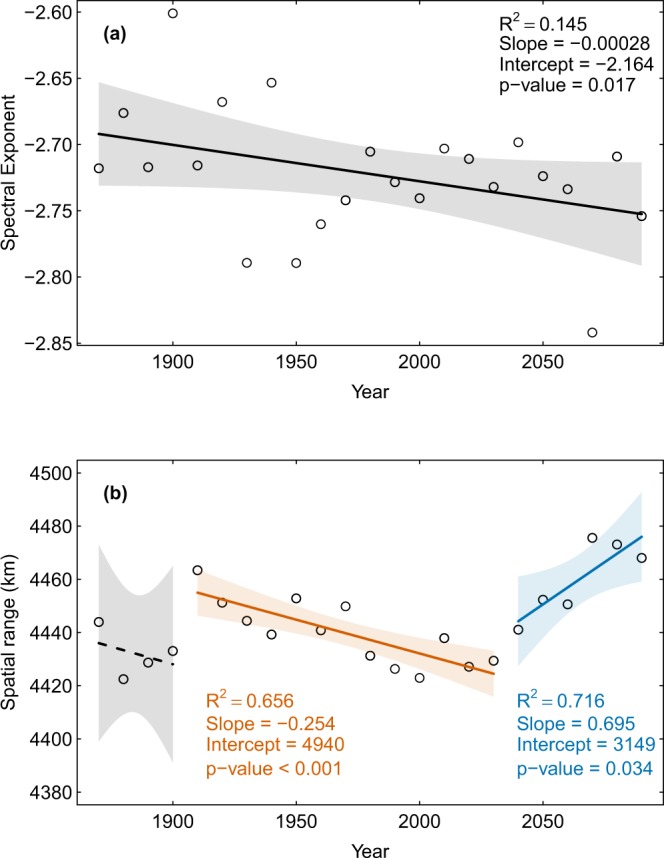
Changes in the temporal and spatial autocorrelation of the multimodel mean temperature obtained via simple regression. (**a**) Temporal autocorrelation was quantified via the spectral exponent, with more negative values indicating greater autocorrelation. (**b**) Spatial autocorrelation was quantified via the spatial range, which measures the geographical distance at which temperatures become decorrelated. Trend lines reflect linear model fits obtained via Generalized Least Squares and shaded regions represent 95% confidence bands. Solid lines indicate statistically significant trends and separate line segments and colors are used to show the breakpoints in the time series. The multimodel mean was computed using 21 GCMs projections and averaged globally over 10-year time windows.

The spectral exponent of the global temperature obtained from the climate multimodel mean becomes more negative over time (“reddening”) indicating increased temporal autocorrelation (Fig. 1). This temporal trend in the multimodel mean holds in terms of its statistical significance (*p*-value < 0.05; Table 1) and sign (Table 2) across multiple time windows ranging from 5 to 10 years. Multiple individual climate models predict a similar increase in the temporal autocorrelation of temperature over time, yielding relatively high model agreement across time windows (Table 2). However, model robustness remains relatively low due to disagreements over the significance of the temporal trend (Table 2). There was no evidence of a breakpoint in the spectral exponent across time windows, indicating a constant rate of increase in temporal autocorrelation over time.

**Table 1:** Temporal and spatial autocorrelation trends obtained via GLS fit for multimodel mean global temperature autocorrelation in 21 GCMs, regressed over time windows from five to ten years.

Years	Spectral exponent			Spatial range (km)		
	Slope	Intercept	*p*-value	Slope	Intercept	*p*-value
5	−0.00021	−2.2995	0.0467	0.11907	4211.5479	0.2848
6	0.00013	−2.963	0.2775	0.09739	4252.6893	0.3085
7	0.000093	−2.9017	0.4887	0.08477	4277.4191	0.4234
8	0.000091	−2.8798	0.4234	0.07445	4296.6776	0.4216
9	0.000196	−3.0943	0.0672	0.09600	4255.9785	0.4154
10	−0.00028	−2.1643	0.0171	0.10216	4243.8615	0.3415

Temporal autocorrelation was quantified using the spectral exponent, with more negative values indicating greater autocorrelation. Spatial autocorrelation was quantified via the spatial range, which measures the geographical distance at which temperatures become decorrelated.

**Table 2:** Agre n global temperature autocorrelation in 21 GCM regressed over time windows ranging from five to ten years for temporal and spatial autocorrelation analyses of the entire time period, and before and after breakpoints identified in Table 3 for spatial autocorrelation.

Years	Spectral exponent		Spatial range (km)							
	Entire time period		Entire time period		Before breakpoint 1		After breakpoint 1		After breakpoint 2	
	Agreement	Robustness	Agreement	Robustness	Agreement	Robustness	Agreement	Robustness	Agreement	Robustness
5	0.43	0.095	0.67	0.62	0.48	0.38	0.71	0.33	0.48	0.38
6	0.48	0.286	0.57	0.62	0.43	0.29	0.71	0.29	0.43	0.29
7	0.38	0.238	0.57	0.43	0.43	0.33	0.67	0.29	0.43	0.33
8	0.52	0.095	0.57	0.43	0.57	0.57	0.71	0.33	0.57	0.57
9	0.38	0.238	0.57	0.43	0.43	0.38	0.71	0.24	0.43	0.38
10	0.38	0.238	0.67	0.33	0.52	0.43	0.67	0.29	0.52	0.43

Temporal autocorrelation was quantified using the spectral exponent, with more negative values indicating greater autocorrelation. Spatial autocorrelation was quantified via the spatial range, which measures the geographical distance at which temperatures become decorrelated. Agreement is defined as the proportion of models whose slopes have the same sign as that of the multimodel mean GLS fit. Robustness is defined as the proportion of models that have the same sign as the multimodel mean GLS fit and a significant trend (*p*-value < 0.05).

### Regional trends in temporal autocorrelation

**Figure 2:**
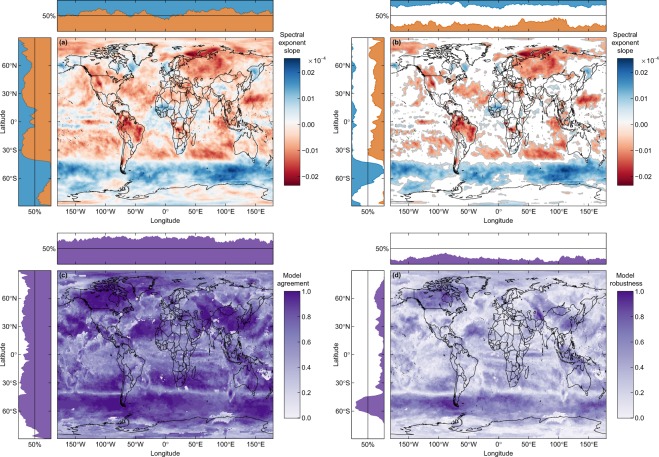
Maps of changes in the temporal autocorrelation of the multimodel mean temperature. Temporal autocorrelation was quantified via the spectral exponent, with more negative values indicating greater autocorrelation. (**a**) Map of the slope obtained by regressing the spectral exponent against time over 10-year periods between 1870 and 2090. Negative (positive) values depicted in red (blue) indicate an increase (decrease) in autocorrelation due to an increase (decrease) in the dominance of lower frequencies. (**b**) Grey contours indicate statistically significant slopes (*p*-value < 0.05). Side plots represent the percentage of geographical locations at each latitude or longitude characterized by an increase in the dominance of lower (red) or higher (blue) frequencies. (**c**) Map of model agreement for the slope of the spectral exponent. Agreement is defined as the proportion of models predicting the same sign for the slope as the multimodel mean, with areas of high agreement being depicted in darker shades of purple. Side plots represent the percentage of geographical locations where model agreement exceed 50% at each latitude or longitude. (**d**) Map of model robustness for the slope of the spectral exponent. Robustness is defined as the proportion of models that agree with the multimodel mean on the sign and the statistical significance of the slope. Side plots represent the percentage of geographical locations where model robustness exceeds 50% at each latitude or longitude.

Although the temporal autocorrelation in temperature increases at the global scale, there is significant geographical variation in both the sign and the strength of the trend (Fig. 2). Temporal autocorrelation in temperature increases most on land in northwestern North America, central South America, northwestern Eurasia, and North and central Africa (Fig. 2a,b). Temporal autocorrelation also increases in the ocean, mostly in the central and southern Atlantic Ocean, the southern Indian Ocean, the Arctic Ocean and the Pacific Ocean. However, temporal autocorrelation decreases almost uniformly across the entire Southern Ocean and West Africa (Fig. 2a,b). Overall, these temporal trends in autocorrelation lead to changes of up to 0.2 units in the spectral exponent from 1870 to 2099 (Fig. S1).

There are no consistent differences in the percentage of geographical locations exhibiting increased vs. decreased temporal autocorrelation of temperature across longitudes. However, there are strong latitudinal patterns, with autocorrelation decreasing most between 70 S and 40 S due to the influence of the Southern Ocean. Autocorrelation increases most in temperate regions due to the effects of both land and sea. In general, model agreement is relatively high across most of the globe, with the notable exceptions of parts of Antarctica and the north Atlantic (Fig. 2c). Model robustness is notably the highest in the Southern Ocean (Fig. 2d). However, robustness is below 50% at other latitudes despite high agreement, which indicates that while individual GCMs are consistent in the trend, there is disagreement about the significance of the trend. Although there is no meaningful change in model agreement and robustness across longitudes, model agreement and robustness vary latitudinally reaching lower values around the South Pole, the equator and the North Pole.

It is important to note that these results are based on the linearly detrended data, which retain seasonal temperature cycles. To determine the robustness of our results to seasonality, we conducted the same analyses on seasonally detrended data (Fig. S2). The results from the seasonally detrended data show an almost universal increase in temporal autocorrelation of temperature across the globe, particularly in the Pacific Ocean, the northern Atlantic Ocean, western North America, South America, western Africa and Australia (Fig. S2). Overall, the predicted trends in the temporal autocorrelation of temperature are stronger and more spatially-uniform when seasonal temperature cycles are removed. This is because seasonal (annual) temperature fluctuations are characterized by high power and occur at relatively low frequencies when using multi-year time windows. Hence, when regressing (log) power against (log) frequency, annual fluctuations in temperature have high statistical leverage on the slope estimate used to quantify the spectral exponent. Removing seasonality, which remains relatively constant over time and has high leverage on the spectral exponent estimate, thus facilitates the detection of temporal trends in autocorrelation due to changes in temperature fluctuations occurring at non-annual frequencies. One can thus think of the trends obtained using the linearly detrended data as a conservative estimate of the expected changes in the temporal autocorrelation of temperature and those obtained using the seasonally detrended data as anti-conservative.

### Global trends in spatial autocorrelation

Spatial autocorrelation in the global multimodel mean temperature measured via the spatial range does not increase significantly over time (Fig. 1). Although the slope relating the spatial range to time is consistently positive across all time windows, it is never significant (Table 1). Among individual climate models, the majority show an increase in the spatial range over time across all time windows, and the trend is robust across 30–60% of individual climate models depending on the time window (Table 2). Despite the lack of a significant relationship at the global scale, piecewise regression shows that across all time windows, there is a breakpoint in the regression of the spatial range against time around year 2030 after which the trend becomes universally significant and positive (Table 2). After the second breakpoint, robustness of the trend in spatial range also increases across all time windows (Table 2). Hence, there is evidence that the spatial autocorrelation of temperature at the global scale is increasing over time after year 2030.

### Regional trends in spatial autocorrelation

**Figure 3:**
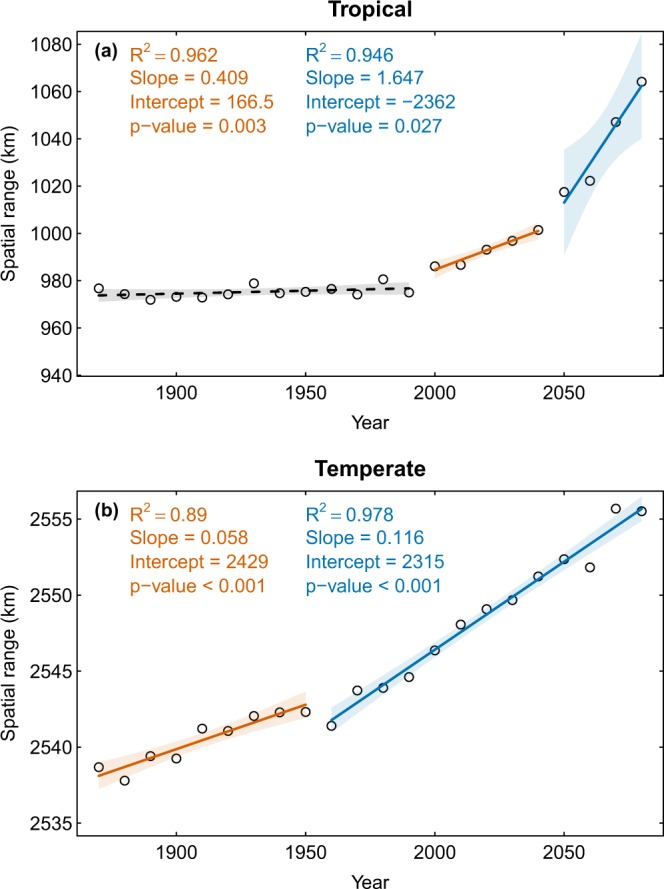
Spatial autocorrelation of the multimodel mean temperature from 21 GCMs, averaged regionally over 10-year time windws. Spatial autocorrelation was quantified via the spatial range, which measures the geographical distance at which temperatures become decorrelated. (**a**) Changes in the spatial range for tropical geographical locations (23.5°S to 23.5°N). (**b**) Changes in the spatial range for temperate geographical locations (23.5°N to 66°N and 66°S to 23.5°S). Trend lines reflect linear model fits obtained via Generalized Least Squares and shaded regions represent 95% confidence bands. Solid lines indicate statistically significant trends, and separate line segments and colors are used to show the breakpoints in the time series.

Spatial autocorrelation in temperature is increasing significantly in temperate (*p*-value < 0.001) and tropical (*p*-value < 0.05) regions over time in the climate multimodel mean (Fig. 3). Across individual climate models, there is strong agreement on the direction of the trend in both temperate and tropical regions (Table S2). Additionally, the trend is highly robust (>90% of individual models) in temperate regions (Table S2). Although spatial autocorrelation is significantly higher in temperate regions than in the tropics, it is increasing at the same rate in both regions (Table S3). The breakpoint in the regression of spatial range against time in the multimodel mean for temperate regions is around 1950, while the breakpoint in tropical regions is around year 2025 (Fig. 3).

## Discussion

Despite substantial variability across the 21 climate models examined, the multimodel mean showed consistent, statistically significant increases in spatial and temporal autocorrelation of temperature at global and regional scales under climate change. Breakpoint analysis of the spatial range of temperature data over time indicated that there was a threshold response in spatial autocorrelation around year 2030 globally and around year 1950 in temperate regions. Furthermore, robustness of the increasing trend and agreement between individual models increased after the breakpoint for spatial autocorrelation globally. Overall, this suggests that the spatial and temporal autocorrelation of temperature are expected to increase under climate change, particularly during the second half of the 21^st^ century.

### Increasing autocorrelation in a warming world

Our results are consistent with other research showing an increase in spatial and temporal autocorrelation of temperature based on weather station data from the second half of the 20^th^ century^2,19^, but inconsistent with one previous study showing a reduction in the temporal autocorrelation of global temperature from weather station data between 1910 and 1990^8^. This discrepancy is likely due to differences in the nature (observations vs. models), spatial resolution and spatiotemporal extent of the temperature records.

For instance, GCMs are simulated at relatively coarse resolutions, are global in scale, longer term, and do not include all of the stochastic factors that influence temperature across temporal and spatial scales. Hence, GCM projections may exhibit an upward bias in the predicted temporal and spatial autocorrelation of temperature relative to observational studies due to the “spatiotemporal smoothing effect” created by their coarser resolution and limited sources of local stochasticity. Although these differences can make comparisons between raw predictions and observations difficult without some form of statistical correction, they should not affect the likelihood of detecting spatiotemporal trends in temperature autocorrelation within GCMs since these trends are measured by making internal comparisons of model predictions.

We suggest that discrepancies between model projections and observational data are more likely due to the large regional differences in the temporal autocorrelation trends seen in both observational studies and GCM projections (Fig. 2). For instance, the analysis of weather data from 1910 to 1990 cited earlier revealed significant regional differences in the temporal autocorrelation of temperature, with Asia and Australia experiencing a significant increase over time, but South America and North America experiencing a significant decrease^8^. Hence, differences in the spatial extent and resolution of observational studies vs. GCM projections could easily lead to conflicting conclusions due to the high degree of regional variation in these trends. Overall, understanding how the nature, resolution and spatiotemporal extent of temperature records interact to influence this discrepancy will be critical in order to more accurately predict the impact of climate change across temporal and spatial scales. Our GCM results echo those of Dillon *et al*.^2^ and Wang and Dillon^19^, who showed an increase in temporal autocorrelation of historical (observed) temperature data from 1961 to 1990 and an increase in spatial homogeneity in weather data between 1960 and 1991. This agreement between our results based on GCM projections and those of Dillon based on observations is consistent with the notion that differences in the spatial resolution and the spatiotemporal extent rather than the nature of the temperature records may have the greatest impact on estimates of temporal and spatial autocorrelation.

Overall, GCM projections suggest that the temporal autocorrelation of temperature will tend to increase more on land than in the sea, with increases occurring in Eurasia, South America, western North America and southeastern Asia, and decreases occurring mostly in the Southern Ocean. This is intriguing because previous studies have shown that the temporal autocorrelation of temperature tends to be lower on land than in the sea^10,20,21^ because heat transfer occurs over longer timescales in the ocean and thus results in greater system “memory”. Taken together, these results suggest that the disparities in the temporal autocorrelation of temperature between land and sea are predicted to decrease over time at the global scale under climate change.

Differences in the spatial autocorrelation of temperature in tropical vs. temperate regions are consistent with expectations from the physical characteristics of the regions. The significant difference between temperate and tropical regions in the mean scale of spatial autocorrelation (i.e., spatial range) is expected because the tropics are much smaller in area than the temperate regions. There is clear evidence of nonlinearity in the spatial data analyzed, particularly in the spatial range of global temperature. Our exploration of the nonlinearities in the global trends via piecewise regression revealed the presence of a quasi-universal breakpoint around the year 2030 across climate models, after which the spatial autocorrelation of temperature increased significantly over time. This lends support for the existence of a “tipping point” in the effect of carbon emissions on the spatial structure of temperature, with time acting as a proxy^22–27^. Although the tipping point in the spatial range occurred around year 2030 in tropical regions, our analyses suggest that in temperate regions the threshold occurred during the 20^th^ century, around the time that the historical effects of anthropogenic climate change became apparent^28^. Overall, this suggests that climate change may thus increase the spatial autocorrelation of temperature at an accelerating rate over the course of the 21^st^ century.

### Ecological impacts of environmental homogenization

Under climate change, our results show that the spatial and temporal autocorrelation of temperature are expected to increase at a potentially accelerating pace over time. This will promote the homogenization of the world, with equator-like conditions spreading geographically to higher latitudes and leading to concomitant changes in the composition of ecological communities^29–31^. This is expected to lead to greater spatial and temporal homogeneity (i.e., due to increased spatial and temporal autocorrelation in temperature).

The ecological consequences of increased homogeneity in the environment have been documented in a number of studies. Increased spatial and temporal homogeneity in environmental variables has been linked to increased extinction risk in populations^15,17,32^. Increased temporal autocorrelation has been shown to promote extinction risk in red-shifted, slow growing species that do not exhibit overcompensatory density-dependence or high sensitivity to environmental stochasticity^8,9^. Indeed, under prolonged exposure to poor environmental conditions, these slow-growing and red-shifted species will see their numbers dwindle and will thus be unable to recover fast enough when conditions improve. Hence, increasing the extent of poor environmental conditions by increasing temporal autocorrelation will erode temporal refugia and prevent temporal rescue effects. Since most animal populations appear to be red-shifted^33,34^, extinction risk is likely to rise due to the increased temporal autocorrelation of temperature under climate change.

Additionally, greater spatial homogeneity in environmental variables such as temperature may disrupt spatial rescue effects by destroying the spatial heterogeneity needed in order for source-sink dynamics to occur^16,35^. Overall, the simultaneous erosion of temporal and spatial refugia under climate change has the potential to promote extinction risk. Mitigating the impacts of these trends in the spatiotemporal properties of temperature is thus critical in order ensure the persistence of complex and interconnected ecosystems in an increasingly homogeneous world.

## Methods

### Data acquisition and availability

Daily surface (air) temperature (variable ‘tas’) between 1871 and 2099 was extracted from 21 General Circulation Model (GCM) under the ‘business-as-usual’ representative concentration pathway (RCP8.5) from the Coupled Model Intercomparison Project Phase 5 (CMIP5^36^; for list of General Circulation Models see Appendix). These 21 GCMs were selected because they included predictions of surface (air) temperature variable at daily temporal scales. All models were standardized to a common 1° × 1° spatial grid and a standard calendar that accounted for leap years. Temperature values above 60 °C were removed as outliers from individual models prior to conducting our analyses.

### Quantifying temporal autocorrelation

The temporal autocorrelation of daily temperature from each model was determined by calculating the spectral exponent. Daily temperature over the entire time period of the model was linearly interpolated at each geographical location to fill in the gaps for models with missing values due to leap years. The interpolated temperature values were then linearly detrended prior to analysis by extracting the residuals from a simple regression relating daily temperature to time at each geographical location over the entire time series. Spectral analysis was then conducted using periodograms computed via fast Fourier transforms in order to determine the variability (power) associated with each frequency in the interpolated and linearly detrended temperature time series^37^. The slope of the linear regression relating the log-transformed power to the log-transformed frequency was used to calculate the spectral exponent^8,9,33^. More negative values of the slope indicate greater temporal autocorrelation (i.e., greater “memory”), with most of the variation in the time series being driven by low frequencies.

To determine the robustness of our results to seasonal variability, we also performed spectral analysis on the seasonally detrended data. To do so, we computed the within-year temperature profile at each geographical location by averaging daily temperatures across the entire time series. We then subtracted the average within-year temperature profile from the daily temperatures at each corresponding geographical location to obtain the seasonally detrended data^10^. Although there were some predictable quantitative differences between the spectral analysis results obtained from the seasonally detrended vs. linearly detrended data, which we discussed at length in the results section, the overall qualitative trends remained largely the same.

### Quantifying spatial autocorrelation

The spatial autocorrelation of temperature over each time period was determined using (semi)variograms to estimate the spatial range, which corresponds to the lag distance (measured in kilometers) at which the semivariance of temperature begins to plateau. By quantifying the geographical distance at which temperatures become uncorrelated, the spatial range can thus be used to represent the spatial scale of autocorrelation. The spatial range was estimated by fitting a Gaussian model to the empirical variogram^38,39^. We also computed the spatial range of temperature in the tropics (23.5°S to 23.5°N) vs. temperate regions (66°S to 23.5°S and 23.5°N to 66°N) and regressed them against time in order to determine whether there were any regional differences in the temporal trends of spatial autocorrelation.

### Quantifying spatial and temporal trends

A Generalized Least Squares (GLS) analysis was used to regress the spectral exponent (temporal autocorrelation) and spatial range (spatial autocorrelation) against time for each of the 21 GCM models as well as the multimodel mean. GLS was used in order to account for heteroscedasticity and autocorrelation of residuals in the spatial and temporal temperature data. To assess model agreement and robustness of trends, we calculated respectively the proportion of models that agreed on the direction of the trend and the proportion of models that both agreed on the direction of trend and were statistically significant (*p*-value < 0.05) as determined by the GLS fits^2,40^. We conducted the spatial and temporal analyses on daily temperature in 10-year time windows between 1871 and 2099 for the global dataset. Each metric of autocorrelation, the spectral exponent or spatial range, was calculated for each time window, and GLS was used to regress the autocorrelation values for each time window against time. We also conducted an extensive sensitivity analysis to determine the robustness of all of our results to different time windows ranging in size from 5 to 10 years (see Tables 1–3).

**Table 3:** Breakpoint analysis for multimodel mean global temperature autocorrelation of 21 GCMs, regressed over time windows from five to ten years.

	Spatial range (km)							
	Before break 1			After break 1			After break 2	
Years	Intercept	Slope	Break year	Intercept	Slope	Break year	Intercept	Slope
5	5020.71	−0.3119	1905	**4996.19**	**−0.2824**	2030	**2649.64**	**0.8773**
6	4861.14	−0.2271	1906	**5013.94**	**−0.3006**	2026	**2646.86**	**0.8776**
7	4876.04	−0.2351	1905	**5014.29**	**−0.2915**	2017	**2446**	**0.9765**
8	4949.48	−0.2744	1902	**4970.92**	**−0.2697**	2030	**2713.35**	**0.8468**
9	5459.62	−0.5459	1897	**4905.08**	**−0.2365**	2014	**2479**	**0.9608**
10	4931.18	−0.2648	1900	**4939.99**	**−0.2539**	2030	**3148.37**	**0.6349**

Bolded coefficients are statistically significant (*p*-value  < 0.05). Spatial autocorrelation was quantified via the spatial range, which measures the geographical distance at which temperatures become decorrelated.

### Quantifying tipping points in spatial and temporal trends

Breakpoint analysis was used to detect any tipping points or significant changes in the linear trends over time in both the temporal and spatial patterns of autocorrelation via piecewise regression^41–44^. This was done using the R package ‘strucchange’, which uses a non-parametric method to estimate the response function and its derivatives, and how that response function changes across the range of the explanatory variable (i.e., time in this case; 44). Function ‘breakpoints’ was used to determine breakpoints in spatial and temporal trends, which identifies the change point by fitting degree 1 splines (piecewise linear functions) with zero through five knot points (change or breakpoint linking the two piecewise functions) of unknown locations^44^. Results were robust to other non-parametric breakpoint analysis methods implemented in R packages ‘SiZer’ and ‘changepoint’^45,46^. Model selection using the small sample size corrected Akaike Information Criterion (AIC_C_) was used to find the optimal number of breakpoints (ref.^47^; see Table S4).

## Electronic supplementary material

Supplementary Information
