# Supplementary material for: Increased spatial and temporal autocorrelation of temperature under climate change
Source: Sci Rep. 2018 Oct 4;8:14850. doi: 10.1038/s41598-018-33217-0 (PMC6172201; doi:10.1038/s41598-018-33217-0)
Supplement: Supplementary file 1 — Supplementary Information [file 41598_2018_33217_MOESM1_ESM.pdf]

# Increased spatial and temporal autocorrelation of temperature under climate change

Grace Di Cecco*[1] and Tarik C. Gouhier[2]

[1] Northeastern University, Department of Biology, 360 Huntington Ave., Boston, MA 02115 dicecco.g@husky.neu.edu
[2] Northeastern University Marine Science Center, 430 Nahant Rd., Nahant, MA 01908, t.gouhier@neu.edu

## Supplementary Information

**Table S1**: Names of the 21 General Circulation Models under CMIP5 used in the study

CMCC-CESM
CMCC-CM
CMCC-CMS
CNRM-CM5
GFDL-CM3
GFDL-ESM2G
GFDL-ESM2M
HadGEM2-AO
HadGEM2-CC
HadGEM2-ES
inmcm4
IPSL-CM5A-LR
IPSL-CM5A-MR
IPSL-CM5B-LR
MIROC5
MIROC-ESM-CHEM
MIROC-ESM
MPI-ESM-LR
MPI-ESM-MR
MRI-CGCM3
MRI-ESM1

**Table S2**: Model agreement of GLS fit for multimodel mean temperature spatial autocorrelation of 21 GCMs averaged by temperate and tropical regions, regressed over ten-year time windows. Spatial autocorrelation was quantified via the spatial range, which measures the geographical distance at which temperatures become decorrelated. Agreement is defined as the proportion of models whose slopes have the same sign as that of the multimodel mean GLS fit. Robustness is defined as the proportion of models that agree on the sign and the statistical significance of the slope ($p$-value < 0.05).

| | Spatial range (km) | |
|---|---|---|
| Region | Agreement | Robustness |
| Tropical | 0.90 | 0.33 |
| Temperate | 0.95 | 0.90 |

**Table S3**: ANCOVA table for GLS fit of the spatial autocorrelation obtained from the multimodel mean temperature averaged by tropical and temperate regions of 21 GCMs and regressed over ten-year time windows. Spatial autocorrelation was quantified via the spatial range, which measures the geographical distance at which temperatures become decorrelated.

| | Spatial range (km) | | |
|---|---|---|---|
| Effect | DF | F-value | *p*-value |
| Year | 1 | 71952.328 | 3.81E-68 |
| Region | 1 | 6368.262 | 1.31E-46 |
| Year x Region | 1 | 3.995 | 0.0525 |

**Table S4.** Model selection of breakpoint models for the spatial autocorrelation observed at global and regional scales was conducted using $AIC_C$. Spatial autocorrelation was quantified via the spatial range, which measures the geographical distance at which temperatures become decorrelated. Models were interpreted as better fits if $\Delta AIC_c > 2$.

| Number of breaks | Global $AIC_C$ | Tropical $AIC_C$ | Temperate $AIC_C$ |
|---:|---:|---:|---:|
| 0 | 193.70 | 187.84 | 80.03 |
| 1 | 184.60 | 144.57 | 57.75 |
| 2 | 186.28 | 136.50 | 69.51 |
| 3 | 203.03 | 153.65 | 91.21 |
| 4 | 238.17 | 200.99 | 141.36 |
| 5 | 338.87 | 351.38 | 291.50 |

**Figure S1**: Map of the difference in the temporal autocorrelation observed over 10-year periods at the end of the 21$^{st}$ century (2080-2090) vs. the end of the 19$^{th}$ century (1870-1880). Temporal autocorrelation was measured using the spectral exponent, with more negative values indicating greater autocorrelation. Negative (positive) values depicted in red (blue) indicate an increase (decrease) in autocorrelation due to an increase (decrease) in the dominance of lower frequencies. Side plots represent the percentage of geographical locations at each latitude or longitude characterized by an increase in the dominance of lower (red) or higher (blue) frequencies. A Wilcoxon rank sum test indicates a significant decrease in the median spectral exponent across all locations from 1870-1880 to 2080-2090 ($p$-value $< 10^{-16}$).

**Figure S2**: Maps of changes in the temporal autocorrelation of the multimodel mean temperature calculated using seasonally detrended daily temperature. Temporal autocorrelation was quantified via the spectral exponent, with more negative values indicating greater autocorrelation. (a) Map of the slope obtained by regressing the spectral exponent against time over 10-year periods between 1870 and 2090. Negative (positive) values depicted in red (blue) indicate an increase (decrease) in autocorrelation due to an increase (decrease) in the dominance of lower frequencies. (b) Grey contours indicate statistically significant slopes ($p$-value $< 0.05$). Side plots represent the percentage of geographical locations at each latitude or longitude characterized by an increase in the dominance of lower (red) or higher (blue) frequencies. (c) Map of model agreement for the slope of the spectral exponent. Agreement is defined as the proportion of models predicting the same sign for the slope as the multimodel mean, with areas of high (low) agreement being depicted in red (blue). Side plots represent the percentage of geographical locations at each latitude or longitude characterized by model agreement above

(red) or below (blue) 50%. (d) Map of model robustness for the slope of the spectral exponent. Robustness is defined as the proportion of models that agree with the multimodel mean on the sign and the statistical significance of the slope. Side plots represent the percentage of geographical locations at each latitude or longitude characterized by model robustness above (red) or below (blue) 50%.

**Figure S1**

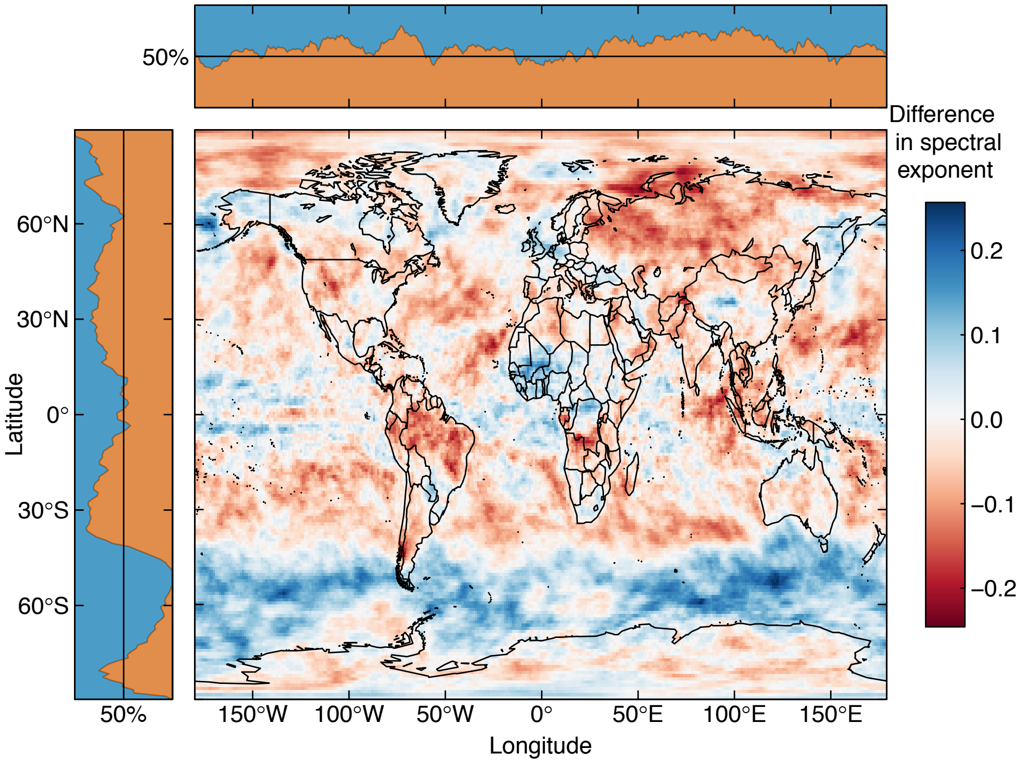
50%
60°N
30°N
0°
30°S
60°S
Latitude
50%
150°W
100°W
50°W
0°
50°E
100°E
150°E
Longitude
Difference in spectral exponent
0.2
0.1
0.0
−0.1
−0.2

# Figure S2

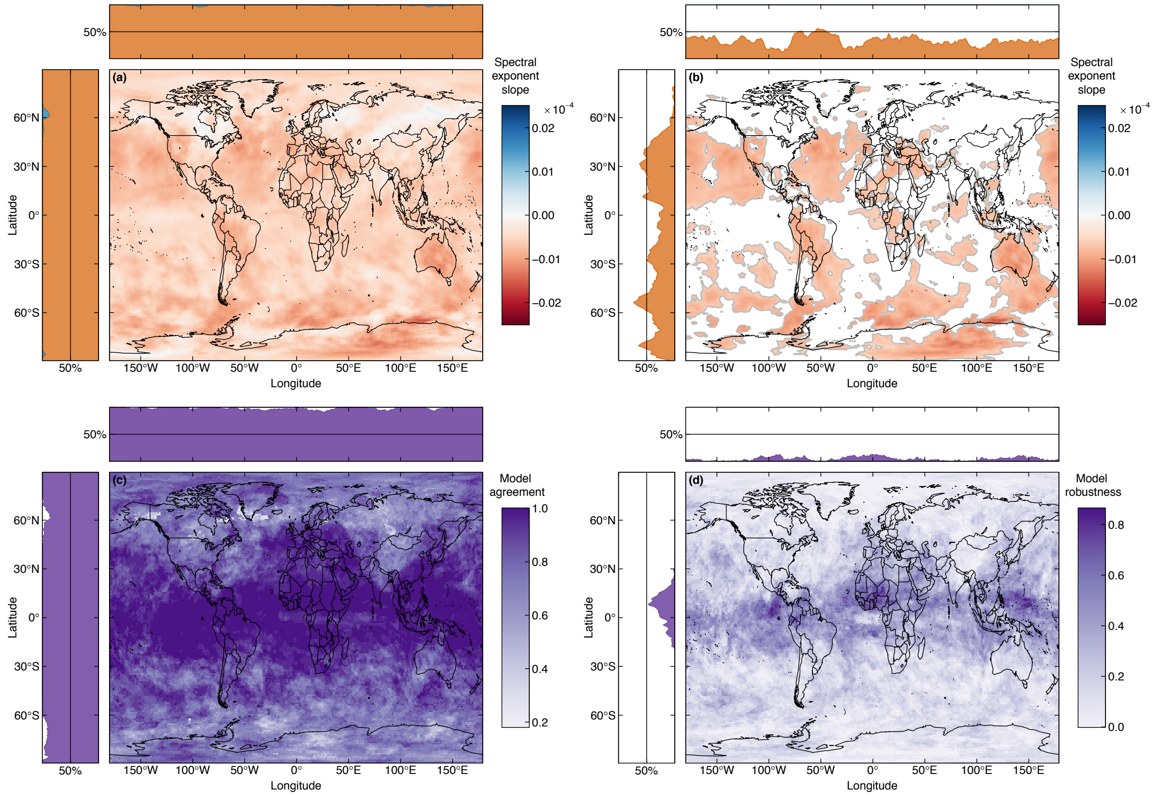

(a)
(b)
(c)
(d)
50%
Latitude
Longitude
60°N
30°N
0°
30°S
60°S
150°W
100°W
50°W
50°E
100°E
150°E
Spectral exponent slope
× 10^-4
0.02
0.01
0.00
−0.01
−0.02
Model agreement
1.0
0.8
0.6
0.4
0.2
Model robustness
0.0
